# Systemic Control of Cell Division and Endoreduplication by NAA and BAP by Modulating CDKs in Root Tip Cells of *Allium cepa*

**DOI:** 10.1155/2014/453707

**Published:** 2014-05-18

**Authors:** Jigna G. Tank, Vrinda S. Thaker

**Affiliations:** Department of Biosciences, Centre for Advanced Studies in Plant Biotechnology and Genetic Engineering, Saurashtra University, Rajkot Gujarat 360 005, India

## Abstract

Molecular mechanism regulated by auxin and cytokinin during endoreduplication, cell division, and elongation process is studied by using * Allium cepa roots* as a model system. The activity of CDK genes modulated by auxin and cytokinin during cell division, elongation, and endoreduplication process is explained in this research work. To study the significance of auxin and cytokinin in the management of cell division and endoreduplication process in plant meristematic cells at molecular level endoreduplication was developed in root tips of * Allium cepa* by giving colchicine treatment. There were inhibition of vegetative growth, formation of c-tumor at root tip, and development of endoreduplicated cells after colchicine treatment. This c-tumor was further treated with NAA and BAP to reinitiate vegetative growth in roots. BAP gave positive response in reinitiation of vegetative growth of roots from center of c-tumor. However, NAA gave negative response in reinitiation of vegetative growth of roots from c-tumor. Further, CDKs gene expression analysis from normal, endoreduplicated, and phytohormone (NAA or BAP) treated root tip was done and remarkable changes in transcription level of CDK genes in normal, endoreduplicated, and phytohormones treated cells were observed.

## 1. Introduction

Endoreduplication cycle is believed to be the switch between cell proliferation and cell differentiation during the developmental stages [1]. The timing of endocycle onset is crucial for correct development programs because polyploidization is linked with cessation of cell division and initiation of differentiation [2]. It is a very common process in plants, frequently associated with differentiation pathways [3]. There is a strong correlation between endoreduplication and cell differentiation. Often the switch from cell proliferation to differentiation is marked by the onset of endoreduplication [4]. The switch from proliferation to differentiation often coincides with the switch from mitotic to endocycles as observed during hypocotyl elongation, trichome growth, and flower and leaf development [5, 6]. It is essential for normal development and physiology in different organisms. For example, endoreduplication occurs during early growth prior to photosynthesis, when the young hypocotyl emerges from the soil. This rapid growth is accomplished through endoreduplication [7]. Endoreduplication associated growth is usually confined to specialized cell types that perform specific biological functions [8]. It occurs only in specific type of tissues where cells should be differentiated into leaf, stem, flower, and root. Hypocotyl cells [9], trichomes [10], leaf pavement cells [11], and developing endosperm of seeds [12] are cells and tissues which undergo endoreduplication before differentiation. Endoreduplication in plants most commonly occurs in tissues that develop mass quickly and have high metabolic activity [13].

Therefore, in the present studies, developing roots of* Allium cepa* were taken as experimental model to study regulation of cell cycle and endoreduplication at molecular level. This plant is an ideal model system for investigating the relationship between cell division and endoreduplication process, as root tips and intercalary meristems of monocotyledons grow fundamentally linear and growth occurs in a well-defined region. In such a linear system, by adopting the cellular view, an organs growth can be determined at a steady-state rate.* Allium cepa* roots were used to study plant cell proliferation and endoreduplication at molecular level due to its relatively simple structure and distinct regions of meristem, elongation, and mature zones. When cells leave the meristematic zone, they enter the elongation zone. Here, they no longer divide but continue to elongate, resulting in a rapid increase in length as a function of position. Basal to the elongation zone, cells are of constant size and considered mature. After maturation they undergo differentiation [14].

Endoreduplication was developed in the roots of* Allium cepa *by using colchicine, as it binds with *β*-tubulin [15] and inhibits microtubule polymerisation, which blocks mitosis [16]. It has indirect role in chromosome doubling by endoreduplication, by affecting the levels of undegradable cyclin B-like proteins [17]. Weingartner et al. [18] showed that endoreduplication and polyploidy occur in cells expressing undegradable cyclin B. Sadhan and Sibdas [19] also showed that the level of cyclin B proteins remained high in colchicine arrested metaphase cells of* Allium cepa*. This suggests that colchicine indirectly stimulates endoreduplication in* Allium cepa* meristematic cells by increasing level of cyclin B proteins.

Further, CDKs (CDKA;1, CDKA;2, CDKB2;1, CDKB2;2, CDKD1;1, and CDKD1;3) gene expression analysis was done from the three different zones (apical zone, elongation zone, and mature zone) of normal and endoreduplicated* Allium cepa* root using RT-PCR. To study transcription level of CDKs genes during mitotic cell division and endoreduplication process at molecular level, cyclin dependent kinases (CDKs) were selected as they are the major regulators of the eukaryotic cell cycle. They are assumed to control cell differentiation and proliferation in response to phytohormonal signals [20].

Phytohormone (NAA or BAP) treatment was given to endoreduplicated roots to reinitiate cell division (which was inhibited by colchicines) in meristematic cells of root tip. Auxin (NAA) and cytokinin (BAP) were selected as they control the most basic physiological processes in plants such as cell division, cell elongation, polarity, and differentiation [21, 22]. They endogenously exert a sequential and restricted control on the cell cycle [23]. They act at multiple levels affecting transcription of CDKs. Their altered balance is required at specific points of the cell cycle to progress from one phase to another [24]. BAP treatment showed increase in growth of endoreduplicated roots and initiated cell division in meristematic cells of root tip after 48 h. However, NAA treatment showed negative response. Hence, CDKs gene expression analysis from phytohormones (NAA or BAP) treated root tips was carried out to observe transcription level of CDKs during these stages.

## 2. Results

### 2.1. Morphological and Cytological Changes in Root Tip Cells of* Allium cepa *after Exogenous Colchicine Treatment

**Figure 1:**
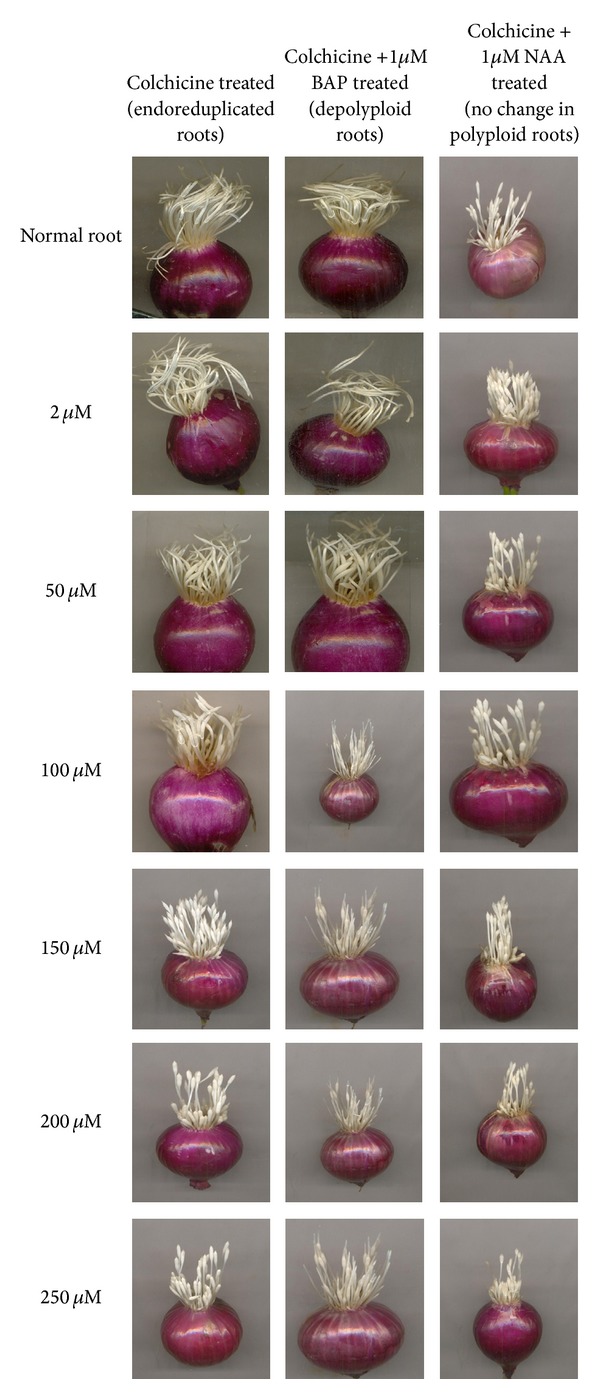
Changes in* Allium cepa *roots growth after colchicine, BAP and NAA treatment.

**Figure 2:**
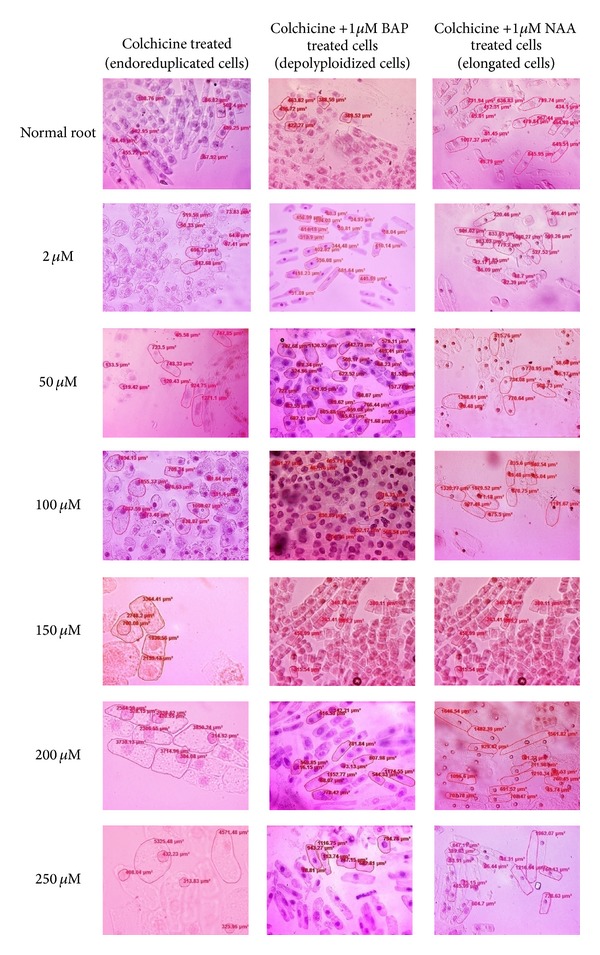
Changes in cell and nucleus size of* Allium cepa *root tip meristematic cells after colchicine, NAA and BAP treatment.

Different concentrations of colchicine (2 *μ*M, 50 *μ*M, 100 *μ*M, 150 *μ*M, 200 *μ*M, and 250 *μ*M) were used to induce endoreduplication in root tips cells of* Allium cepa.* There was remarkable inhibition in growth of roots and c-tumor formation was observed at the tip of roots (Figure 1; see Supplementary Figure 10 in Supplementary Material available online at http://dx.doi.org/10.1155/2014/453707). Cytological analysis showed that the cells of root tip were endoreduplicated and were arrested at metaphase. There was an increase in cell and nucleus size after colchicine treatment (Figure 2). DNA was isolated from normal and endoreduplicated root tips of* Allium cepa* to determine the change in DNA level due to endoreduplication process induced within root tip cells (data published [25]).

### 2.2. Morphological and Cytological Changes in Endoreduplicated Root Tip Cells of* Allium cepa* after Exogenous NAA Treatment

Colchicine treatment inhibited cell division and further growth of roots. To reinitiate growth and cell division in roots of* Allium cepa*, naphthyl acetic acid (NAA) treatment was given to roots. Different concentrations of NAA (1 *μ*M, 50 *μ*M, 100 *μ*M, and 250 *μ*M) were used to observe dose dependent response. However, NAA was not able to give positive response in initiating cell division and growth of roots (Figures 1 and 2 and Supplementary Figure 11).

### 2.3. Morphological and Cytological Changes in Endoreduplicated Roots of* Allium cepa* after Exogenous BAP Treatment

Exogenous BAP treatment showed drastic changes in endoreduplicated root tips. There was an increase in growth of roots observed after 24 h of BAP treatment in all concentrations (1 *μ*M, 50 *μ*M, 100 *μ*M, and 250 *μ*M) (Supplementary Figure 12). After 24 h tip of c-tumor showed growth; this increased with increase in incubation time from 24 h to 120 h (Supplementary Figure 10). There was gradual increase in length of roots with increase in incubation time from 24 h to 120 h (Figure 1). Cytological analysis showed that endoreduplicated cells which were arrested in metaphase started anaphase and cytokinesis process after 24 h of BAP treatment. In all concentrations of colchicine tested, there was a decrease in cell size and nucleus size. All mitosis phases (prophase, metaphase, anaphase, and telophase) were observed in root tip cells after BAP treatment (Figure 2).

### 2.4. Gene Expression Analysis of CDKs Genes

**Figure 3:**
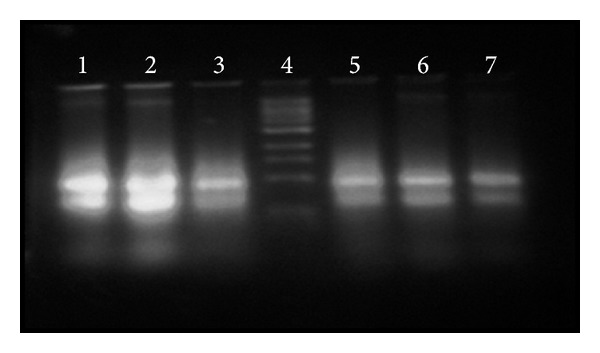
Bands of total RNA on 3% denaturing agarose gel (Lanes 1, 2, and 3 show total RNA isolated from apical, elongated, and mature zone of endoreplicated root. Lane 4 shows Riboruler and Lanes 6, 7, and 8 show total RNA isolated from apical, elongated, and mature zone of normal root).

**Figure 4:**
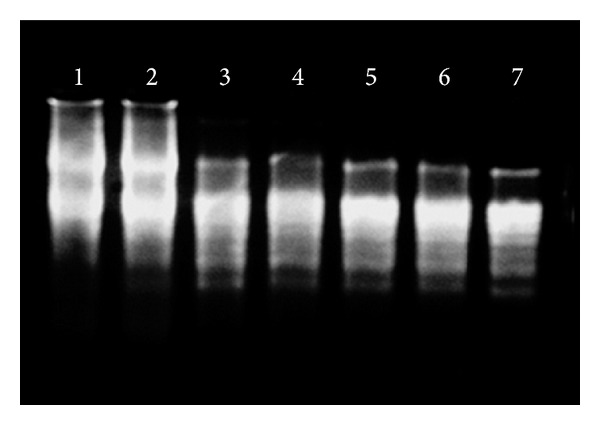
Bands of cDNA synthesized using high capacity cDNA synthesis kit (Lanes 1 and 2 show RNA bands and Lanes 3, 4, 5, 6, and 7 show cDNA bands).

To study the basic phenomenon responsible for regulation of cell division, endoreduplication, elongation, and differentiation processes in plants at molecular level during growth of* Allium cepa* roots, quantification of the relative level of transcripts of CDKs genes was done. Normal and endoreduplicated roots of* Allium cepa* were dissected into three zones: (1) apical zone, (2) elongation zone, and (3) mature zone. RNA was isolated from these zones of root. RNA concentration and purity were measured. There was remarkable increase in the RNA level in endoreduplicated root tips as compared to normal root tips. Result was confirmed by comparing RNA content obtained from A_260 nm_ with the agarose gel electrophoresis. RNA band intensity was high in the sample of endoreduplicated cells as compared to normal cells (Figure 3). Normal and endoreduplicated RNA samples having purity of 1.95 were selected and cDNA was synthesized from them (Figure 4). cDNA samples were used for CDKs gene expression analysis. 

**Figure 5:**
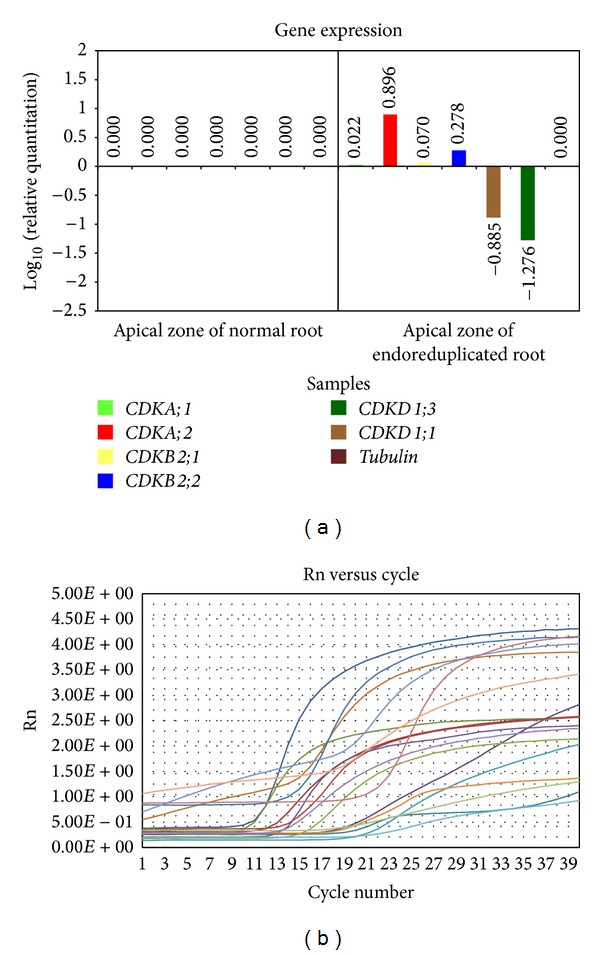
Graph of gene expression level (a) and amplification plot (b) of CDKs genes in apical zone of normal and endoreduplicated* Allium cepa *root.

*(1) Expression of CDKs Genes in Apical Zone of Normal and Endoreduplicated Roots*. Quantification of the relative level of transcripts of CDKs genes (*CDKA:1, CDKA;2, CDKB2;1, CDKB2;2, CDKD1;1, *and* CDKD1;3*) was done. The expression level of* CDKA;1* and* CDKB2;1* was low in endoreduplicated root tip cells but somewhat higher than normal root tip cells. Expression level of* CDKA;2* remained much higher in endoreduplicated cells than the normal cells. However,* CDKD1;1* and* CDKD1;3* genes showed expression level much lower in endoreduplicated root tip cells than the normal root tip cells (Figure 5). 

**Figure 6:**
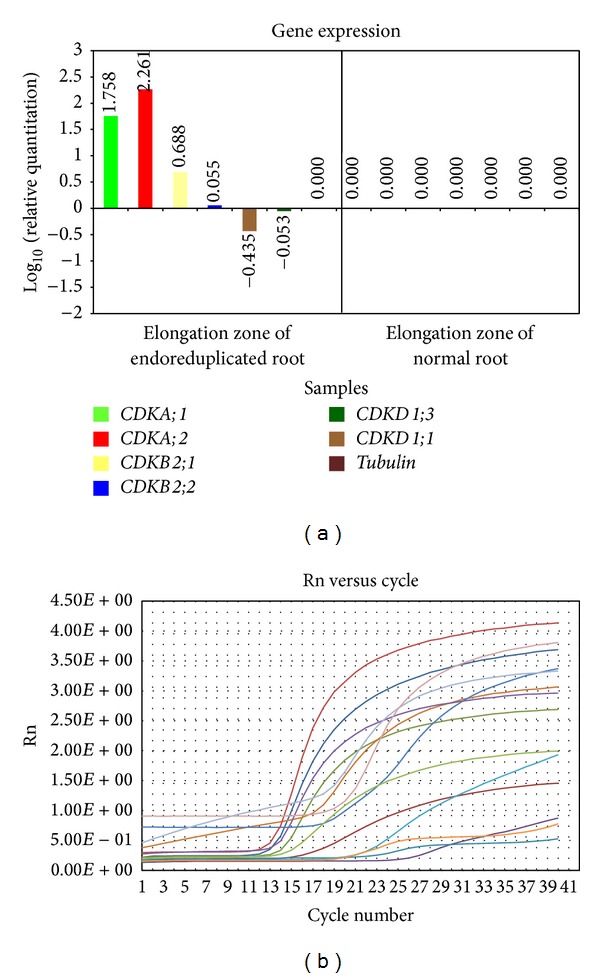
Graph of gene expression level (a) and amplification plot (b) of CDKs genes in elongation zone of normal and endoreduplicated* Allium cepa* root.

*(2) Expression of CDKs Genes in Elongation Zone of Normal and Endoreduplicated Roots*. In elongation zone cells, transcription level of CDK genes differed much as compared to apical zone cells of root. There was a vast difference in expression of all CDK genes. Transcription level of* CDKA;1* and* CDKA;2* gene was higher in endoreduplicated cells as compared to normal cells.* CDKB2;1* was somewhat higher in endoreduplicated cells than normal cells whereas* CDKB2;2* was lower in endoreduplicated cells than normal cells.* CDKD1;1* and* CDKD1;3* were much lower in endoreduplicated cells than in normal cells (Figure 6). 

**Figure 7:**
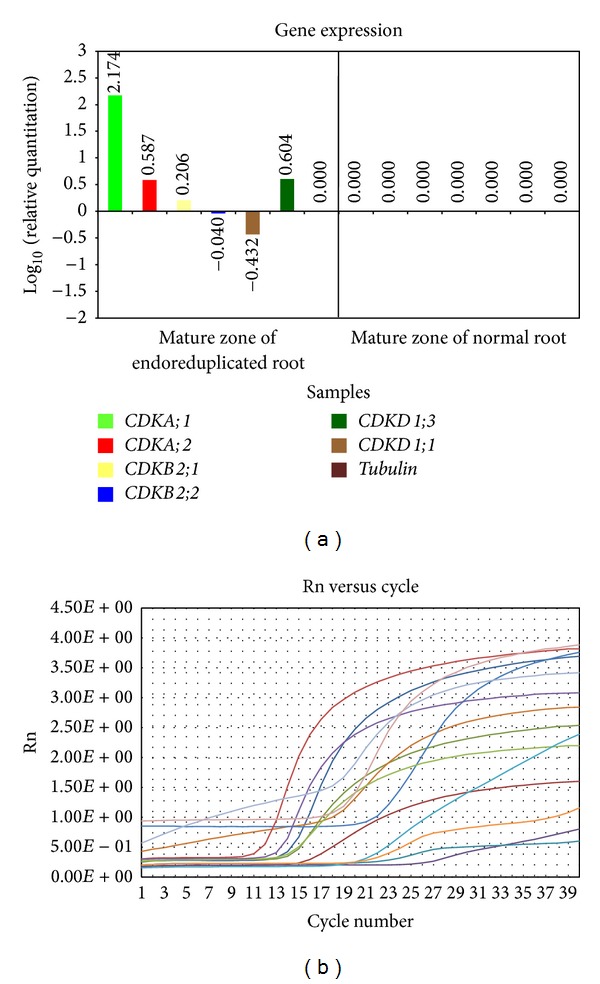
Graph of gene expression level (a) and amplification plot (b) of CDK genes in mature zone of normal and endoreduplicated* Allium cepa* root.

*(3) Expression of CDKs Genes in Mature Zone of Normal and Endoreduplicated Roots*. In mature zone transcription level of* CDKA;1* gene increased much in endoreduplicated cells than in normal cells. The expression of* CDKA;2*,* CDKB2;1,* and* CDKD1;3* gene was somewhat higher in endoreduplicated cells than in normal cells.* CDKB2;2* was lower and* CDKD1;1* was much lower in endoreduplicated cells than normal cells (Figure 7). 

*(4) Expression of CDKs Genes in BAP Treated Endoreduplicated Root Tips of Allium cepa*. CDK gene expression analysis was done from the root tips which showed growth in root length after BAP treatment. This was done to know how BAP diverted arrested cell cycle towards cell division, elongation, and differentiation process. Here root was not dissected into three zones but only tip portion of root which showed growth and development was taken. This was done because only apical meristematic zone cells showed cell division phases in microscope whereas there was no change in elongation and mature zone.

**Figure 8:**
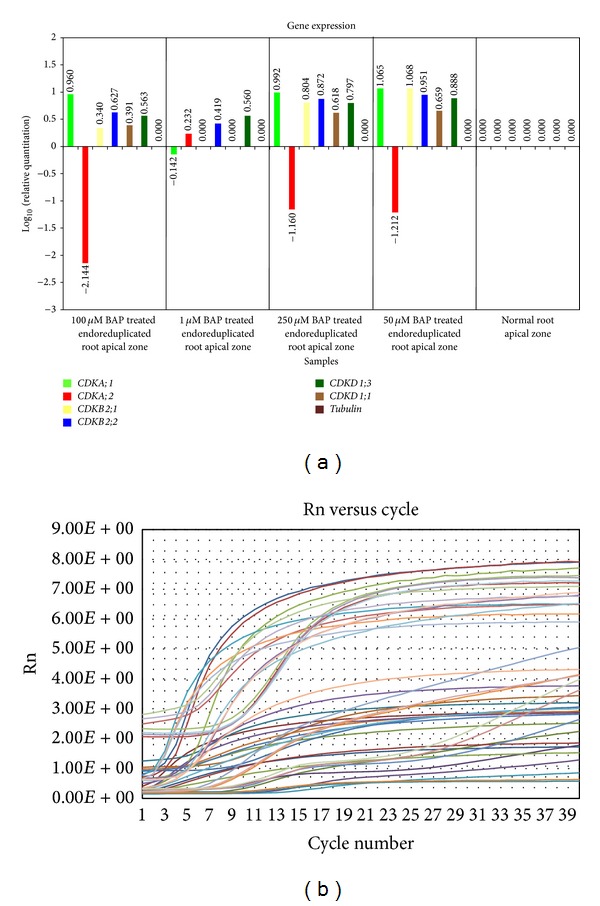
Graph of gene expression level (a) and amplification plot (b) of CDK genes in apical zone of normal and endoreduplicated root after BAP treatment.

Remarkable difference in expression level of CDK genes was observed after BAP treatment to endoreduplicated apical root tip cells of* Allium cepa*. Concentration dependent response of BAP in regulation of CDK genes was observed. Transcription level of* CDKA;1* gene was higher in 50 *μ*M, 100 *μ*M, and 250 *μ*M BAP treated root tips but lower in 1 *μ*M BAP treated root tip cells (Figure 8). In contrast,* CDKA;2* gene expression was much lower in three concentrations of BAP (50 *μ*M, 100 *μ*M, and 250 *μ*M) but moderate in 1 *μ*M BAP treated root tips (Figure 8).* CDKB2;1* gene showed higher expression level in 50 *μ*M, 100 *μ*M, and 250 *μ*M BAP treated root tips, but no expression level was observed in 1 *μ*M BAP treated root tips (Figure 8).* CDKB2;2* gene showed higher expression level in all concentrations of BAP tested (Figure 8).* CDKD1;1* and* CDKD1;3 *were also high in all concentrations of BAP tested. However, expression level of* CDKD1;3* was higher than expression of* CDKD1;1 *(Figure 8). 

*(5) Expression of CDKs Genes in NAA Treated Endoreduplicated Roots*. Since NAA was not able to induce growth in apical root tips of* Allium cepa* transcription level analysis was done from these NAA treated cells. This was done to know why cell division was not induced and what the function of auxin during cell division and differentiation process is, and how it regulates the transcription level of CDK genes at molecular level. Here also root was not dissected into three zones but only tip portion of root was taken.

**Figure 9:**
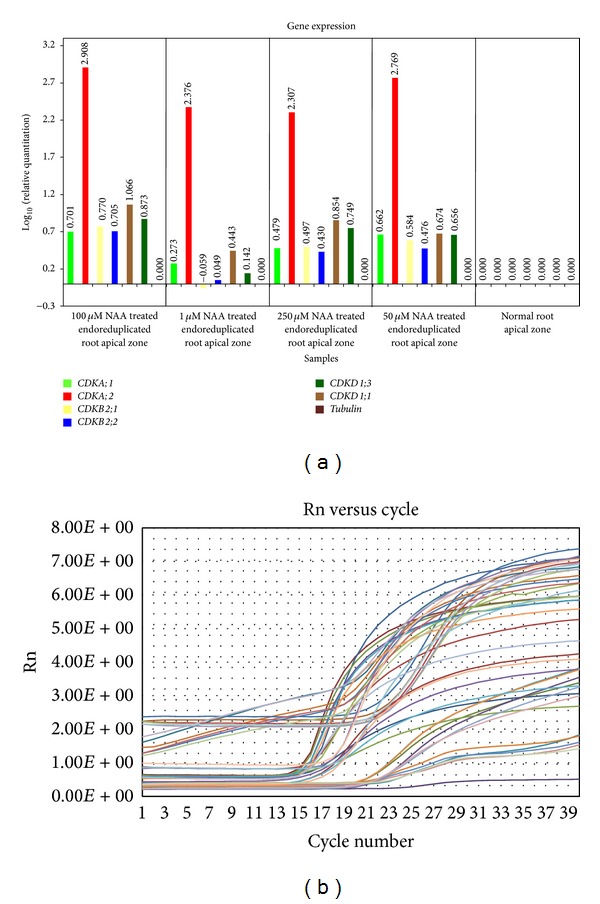
Graph of gene expression level (a) and amplification plot (b) of CDK genes in apical zone of normal and endoreplicated root after NAA treatment.

Endoreduplicated cells were treated with four different concentrations of NAA to know the concentration dependent response of CDK genes. In all the four concentrations of NAA tested* CDKA;2* gene showed much higher expression level than normal and BAP treated cells (Figures 8 and 9). Expression of* CDKA;1* gene was moderately high in all NAA concentrations. But in 1 *μ*M NAA treated root tips it was lower than the other three concentrations (50 *μ*M, 100 *μ*M, and 250 *μ*M) (Figure 9). Also, it was observed that expression level of* CDKA;1* was high in BAP treated cells than NAA treated cells (Figures 8 and 9). Transcription level of* CDKB2;1* was lower in all concentrations of NAA tested than BAP treated root tips (Figures 8 and 9).* CDKB2;2* expression was also lower in all concentrations of NAA tested than in the BAP treated root tips (Figures 8 and 9). However, 1 *μ*M NAA treated root tip showed much lower expression level of CDKB2;2 than the other concentrations of NAA tested (Figure 9). Expression level of CDKD1;1 was higher in NAA treated cells than in BAP treated cells (Figures 8 and 9).

## 3. Discussion

Different CDK genes, namely,* CDKA;1, CDKB2;1, CDKB2;2, CDKD1;1,* and* CDKD1;3* were selected as they have important function in the regulation of cell cycle at G1/S and G2/M transition.* CDKD* was selected because it is a member of a kinase network that regulates CDK activity via a phosphorylation cascade. It activates other plant CDKs by phosphorylating conserved threonine residue in the T-loop region [26]. Further,* CDKA;1, CDKA;2, CDKB2;1,* and* CDKB2;2* genes were selected because, in tomato, the expression of* CDKA;1, CDKA;2, CDKB1;1,* and* CDKB2;1* was detected more readily in organs with actively dividing cells, such as young leaves, roots, and suspension cultured cells. However, relatively low transcript levels of these genes (*CDKA;1, CDKA;2, CDKB2;1,* and* CDKB2;2*) were observed in nondividing tissues such as mature leaves and stems [27, 28]. Similar observations were reported in petunia, maize,* Arabidopsis*, soybean, and alfalfa [29, 30].* CDKA;1* (previously designated CDC2aAt; [27]) in* Arabidopsis* showed kinase activity during S, G2, and M phases of the cell cycle. These data indicated that A-type CDKs regulate both (G1/S and G2/M) transitions. Initiation of the G2/M phase transition requires induction of the B-type cyclins (CYCB) and CDKB gene [31]. CYCB interacts with CDKB to initiate phosphorylation, activate proteins, and express genes required for cytokinesis [32]. In alfalfa cells synchronized with aphidicolin, the* Medsa;CDKB1;1 *(cdc2MsD) gene was expressed earlier in the G2 phase and then the* Medsa,CDKB2;1 *(cdc2MsF) gene was expressed in the late G2/M phase. Zhiponova et al. [33] suggested that Medsa;CDKB2;1 is a mitosis-specific promoter in alfalfa cells. Its transcription level increases at G2/M transition and decreases at G1 phase [34–36].

### 3.1. Expression of CDKs Genes in Apical Zone of Endoreduplicated* Allium cepa* Root Cells

*CDKD1;1* and* CDKD1;3* are CDKs activating kinases and they are involved in the initiation of plant cell cycle and activation of CDKA [36]. Transcription level of these genes in endoreduplicated cells was negative suggesting that their function of initiation of G1/S transition was completed and further endoreduplication process was going on. CDKD1;1 is involved in the control of S phase entry, progression through DNA replication process, and activation of downstream CDKs [37]. Since cells were already endoreduplicated, expression of this gene was negative. CDKD1;3 plays a role in the activation of CDK activity during cell cycle reentry [26]. In present studies since root tip cells were in endoreduplication stage expression of these genes was negative. CDKA;1 gets activated by CDK activating kinases and gives signal to CDKA;2 (previously known as cdc2MsB) to enter G1/S phase [38] and start DNA replication process. Similar results were observed in present studies of endoreduplicated root tip cells (Figure 2). Here, expression level of CDKA;1 gene was somewhat higher than normal because it has role in giving signal to CDKA;2. Expression of CDKA;2 remained much higher in endoreduplicated cells than normal cells suggesting the role of CDKA;2 in DNA replication process (Figure 5). In our studies expression of CDKB2;1 gene was somewhat higher in endoreduplicated cells than normal cells (Figure 5) because it has role in control of G2/M phase transition and mitotic events shown by Umeda et al. [39]. Meanwhile, endoreduplication cells remain in the M phase before cytokinesis; therefore, expression of CDKB2;1 gene was higher in endoreduplicated cells as compared to normal cells (Figure 5). Transcription level of CDKB2;2 was high in endoreduplicated cells because it has role in organization of meristematic cells [36]. This resulted in formation of c-tumor in endoreduplicated root tips of* Allium cepa.*

### 3.2. Expression of CDKs Genes in Elongation Zone of Endoreduplicated* Allium cepa* Root Cells

In morphological analysis there was decrease in length of roots with increase in endoreduplication. In elongation zone, since cells are in elongation process relative gene expression analysis was carried out to know the role of CDKs in this process. Expression of CDKD1;1 and CDKD1;3 genes was negative in elongation zone of endoreduplicated cells (Figure 6) supporting the previous analysis that they have function in initiation of cell cycle and no role in elongation of cells [36]. Expression of CDKA;1 and CDKA;2 remained higher in endoreduplicated cells as compared to normal cells (Figure 6) because endoreduplicated cells continuously remain in S phase inhibiting the mitosis, cytokinesis, and elongation process [8]. Expression of CDKB2;1 remained somewhat higher in endoreduplicated cells compared to normal cell (Figure 6) suggesting that endoreduplicated cells were arrested in G2/M phase. Expression of CDKB2;2 was lower than the CDKB2;1 in endoreduplicated cells (Figure 6) indicating that cells were already arranged in root but, due to increase in cell size because of endoreduplication process, its function is required at low level to arrange them [36].

### 3.3. Expression of CDKs Genes in Mature Zone of Endoreduplicated* Allium cepa* Root Cells

In endoreduplicated cells transcription of CDKD1;3 and CDKA;1 gene increased in maturation zone showing that mature cells are reentering in G1/S phase. Since cells were in S phase, the expression of CDKD1;1 gene remained negative (Figure 7). Expression of CDKA;2 remained somewhat high in mature phase showing that cells are reentering the cell division process (Figure 7). Expression of B2;2 remained negative in mature zone showing that the cells are already organized and its function is not required (Figure 7). Expression of B2;1 remained somewhat higher in endoreduplicated cells than the normal cells showing its function in initiation of S phase (Figure 7). This suggests that endoreduplication process does not allow cells to enter into mature stage. These results correlate with the morphological studies which showed that with endoreduplication there was inhibition in growth of root length (Figure 1).

### 3.4. Expression of CDKs Genes in Phytohormones (Auxin or Cytokinin) Treated Root Tip Cells of* Allium cepa*

Auxins and cytokinins are members of a biochemical network which control the transcription of cell cycle genes [40–42]. In roots, the mitotic to endocycles transition was recently proposed to be regulated by auxin and cytokinin, the combination of low auxin and increased cytokinin facilitating the switch from mitotic cells to endocycle and differentiation [43, 44]. Similar results were obtained in the present studies showing that growth of endoreduplicated roots was sustained again by giving the exogenous treatment of BAP to roots (Figure 1). Exogenous BAP treatment increased concentration of cytokinin in endoreduplicated cells and directed cell cycle towards differentiation process. These resulted in initiation of cell division process in root tip cells and increase in length of roots (Figure 1). Exogenous cytokinin treatment controlled cell cycle of endoreduplicated cells by changing the expression level of CDK genes. The expression level of* CDKA;1, CDKD1;1, CDKD1;3, CDKB2;1, *and* CDKB2;2* gene increased and expression level of CDKA;2 gene decreased after BAP treatment (Figure 8). This suggests that meristematic cells of endoreduplicated root tip were diverted towards G2/M phase transition. Concentration dependent response of BAP in controlling cell cycle at molecular level was also observed. Concentrations of BAP, that is, 50 *μ*M, 100 *μ*M, and 250 *μ*M, were much effective in inducing this process. However, 1 *μ*M BAP treatment was capable enough to induce G2/M process, but it was not sufficient enough to increase the expression level of* CDKA;1, CDKD1;1, CDKD1;3, CDKB2;1, *and* CDKB2;2* genes. Also, it was not able to reduce the expression level of* CDKA;2* gene (Figure 8).

By modulating the levels of cell cycle regulators that are involved in both the mitotic cycle and the endocycle, auxin is likely to play a major role in the regulation of endoreduplication [44]. In the present studies, auxin level was increased in root tip cells by giving exogenous NAA treatment. However, growth of endoreduplicated roots was not sustained. But it changed the transcription level of CDK genes and resulted in diversion of cell cycle towards elongation process. IAA (indoleacetic acid) is known to induce cell enlargement without cell division in tobacco pith explants grown on an agar medium without added cytokinin [45]. Similar results were obtained in the present studies of endogenous zeatin level measurement, which increased gradually in normal root tips with increase in length of roots from 24 h to 120 h. However, it decreased in endoreduplicated root tips where growth of roots was inhibited. It remained stable in endoreduplicated root tips even after NAA treatment. The decrease in zeatin level and increase in NAA level resulted in elongation of endoreduplicated cells (Figure 2). Quélo et al. [46] also showed that mesophyll protoplast derived cells cultured in the presence of NAA (auxin) and BAP (cytokinin) keep on dividing, while elongation and concomitant DNA endoreduplication are induced and maintained in a medium containing only NAA. For cultured tobacco cells, an auxin alone signal induces elongation and DNA endoreduplication, whereas addition of auxin and cytokinin causes the cells to divide actively [47].

Horvath et al. [48] suggested that auxin promotes elongation in nondividing cells. Since cell division was inhibited in root tip cells by using colchicine, NAA treatment induced cell elongation in these endoreduplicated cells. Exogenous application of micromolar auxin stimulates cell expansion in stems [44]. However, the molecular mechanisms underlying these differences in auxin sensitivity remained elusive. We have made an attempt to answer this question by studying the transcription level of CDK genes in root tip cells treated with NAA. CDK genes were selected because they are the key cell cycle regulators at molecular level in plants [13]. Different concentrations of NAA were used to study the dose dependent response of NAA on molecular mechanism of cells. Auxin-dependent cell expansion follows a dose-response curve in which high concentrations are inhibitory [49, 50]. Among the different concentrations of NAA tested higher concentrations (100 *μ*M and 250 *μ*M) were inhibitory after 72 h. Hence, gene expression analysis of CDK genes was done after 48 h of NAA treatment. The expression level of* CDKA;1, CDKD1;1, CDKD1;3, CDKB2;1, *and* CDKB2;2* was moderately high in 50 *μ*M, 100 *μ*M, and 250 *μ*M NAA treated root tips. However, expression level of these genes (*CDKA;1*,* CDKD1;1*,* CDKD1;3*,* CDKB2;1*, and* CDKB2;2*) were low in 1 *μ*M NAA treated root tips. This suggests that 1 *μ*M concentration of NAA was not sufficient to change the expression of CDK genes in root tip cells (Figure 9).

The expression of* CDKA;2* was much higher in all concentrations of NAA treated root tips (Figure 9) because it is involved in DNA replication process [51]. Also, auxin is known to induce both cell enlargement and an increase in nuclear DNA without cell division [52–54]. When auxin is applied to* Prunus armeniaca *(apricot) trees, it provokes an increase in fruit size because of the endoreduplication driven enlargement of mesocarp cell volume [55]. Similarly, in cultured haploid* Petunia hybrid *leaf tissues, auxin treatment induces endopolyploidy by doubling the chromosomenumber [56]. The change in expression of* CDKA;1, CDKB2;1, CDKB2;2, CDKD1;1,* and* CDKD1;3* gene indicates that these genes might be involved in cell elongation process (Figure 9). John et al. [57] showed that, in the elongation zone of pea roots, auxin induces a rise in the transcript of* CDKA*. Cells in this zone have been recently formed and they have* CDKA *mRNA above the basal level found in fully differentiated cells. Yoshizumi et al. [58] suggested that* Arath;CDKB1;1* is involved in regulating directly hypocotyl cell elongation or a specific phase of the cell cycle and/or overall chromosome spatial organization, critical for hypocotyl cell elongation and cotyledon development. Takatsuka et al. [36] suggested that* CDKF;1* targets CDKD that functions in regulating cell division, cell elongation, and endoreduplication during postembryonic development. In wild-type roots, the* CDKD;2* GUS fusion protein was accumulated in the meristematic and elongation zones, but it disappeared in the root tips of the* cdkf;1-1* mutants. Similarly, in our studies, transcription level of* CDKD1;1* gene remained high in cells which were elongated after NAA treatment (Figures 9 and 2). However, the expression level of* CDKD1;3* was lower than the expression level of* CDKD1;1* in these cells. These suggest that* CDKD1;1* is involved in cell elongation process, whereas* CDKD1;3* is involved in cell division process only. Also, it was noticed that expression of* CDKD1;3* was high in BAP treated meristematic cells whose cell size was small (Figures 8 and 2).

## 4. Conclusions

From the above studies it can be concluded that cytokinin (BAP) treatment to endoreduplicated root tips changed the transcription level of various CDK genes in meristematic cells of root. It downregulated the activity of* CDKA;2 *and inhibited DNA replication process in cells. It induced expression of* CDKD1;3* gene with the help of* CDKF* gene, which in turn increased the expression level of* CDKA;1* gene.* CDKB2;1* expression level remained high in BAP treated cells because it allowed cells to proceed from G2 to M phase.* CDKB2;2* gene transcription level remained high because it is involved in organization of meristematic cells. Since roots were growing in length organization of cells in root tissue is required.

Auxin (NAA) treatment to endoreduplicated root tips increased the expression level of* CDKA;2* gene in cells as it is involved in DNA replication process. It downregulated expression of* CDKD1;3* gene and increased expression of* CDKD1;1* gene using* CDKF*. This increased expression of* CDKA;1* gene and diverted cell cycle towards elongation process. Expression level of* CDKD1;3* increased when cell was undergoing cell division process and its expression was downregulated when cell diverted process towards elongation. As the cells moved towards elongation process expression level of* CDKB2;1* became lower than the BAP treated cells as this gene has role in G2/M transition (Figures 8 and 9). Cells were already arranged and there was no increase in length of roots, so activity of* CDKB2;2* was low in these meristematic cells. Hence, this research work elucidates regulation of molecular mechanism by auxin during cell elongation process and by cytokinin during cell division process.

## 5. Materials and Methods

### 5.1. Treatment of Roots with Colchicine (Endoreduplication Inducing Agent)

Young roots of onion were treated with various concentrations of colchicine ranging from 2 *μ*M to 250 *μ*M. Roots were exposed to colchicine treatment for 120 h at room temperature. Morphological and cytological changes in root tips of* Allium cepa* were observed at an interval of 24 h.

### 5.2. Treatment of Roots with Phytohormones

After 120 h of colchicine treatment endoreduplicated root tips were obtained. These root tips were exogenously treated with NAA for 120 h. Similarly, endoreduplicated root tips were exogenously treated with BAP for 120 h. Changes in morphological and cytological characters (length and width of roots, cell and nucleus size of root tip cells) were observed at an interval of 24 h. Roots were treated with different concentrations of NAA or BAP ranging between 1 *μ*M, 50 *μ*M, 100 *μ*M, and 250 *μ*M to study the concentration dependent response of cells during growth and development of roots.

### 5.3. Microscopic Observation for Mitosis

Microscope slides were prepared from normal and treated root tip samples at an interval of 24 h. They were prepared by following the Feulgen staining protocol of Ohri et al. [59]. The slides were analyzed using Axiovision Carl Zeiss microscope. Cell size and nucleus size of root tip cells were measured by using imaging power of Axiovision 4 software.

### 5.4. RNA Isolation

RNA was isolated from normal, endoreduplicated (250 *μ*M colchicine treated for 48 h), and phytohormones treated (1 *μ*M, 50 *μ*M, 100 *μ*M, and 250 *μ*M for 120 h) root tips of* Allium cepa*. 1 gm root tips were homogenized in tri-reagent (0.5 mL) using mortar and pestle. Homogenized samples were allowed to stand for 20 min. at room temperature to ensure complete dissociation of nucleoprotein complexes. Chloroform (0.2 mL) was added per ml of tri-reagent used to all samples and shaken vigorously for 15 seconds. They were allowed to stand for 15 minutes at room temperature. Centrifugation of the resulting mixture was done at 12,000 ×g for 15 minutes at 2–8°C. Centrifugation separated the mixture into 3 phases: a red organic phase (containing protein), an interphase (containing DNA), and a colorless upper aqueous phase (containing RNA). The aqueous phase was transferred to a fresh tube and isopropanol (0.5 mL) was added to it. Mixture was allowed to stand for 10 minutes at room temperature. The precipitated RNA was pelleted by centrifugation at 12,000 ×g for 10 minutes at 2–8°C. RNA pellet was washed by adding 100% ethanol, vortexing the sample, and centrifuging it at 7,500 ×g for 5 minutes at 2–8°C. Further, RNA pellet was air-dried for 5–10 minutes. Appropriate volume of DEPC treated TE buffer was added to the RNA pellet to facilitate dissolution and mixed properly by repeated pipetting with a micropipette at 55–60°C for 10–15 minutes. The concentration and purity of RNA samples were evaluated by measuring the absorbance at 260 nm (A_260_) and 280 nm (A_280_) in a microplate reader (Biotek *μ*Quant). Quality of total RNA was determined by running it on 3% denaturing agarose gel.

### 5.5. cDNA Synthesis from Total RNA

cDNA was synthesized by using high capacity cDNA synthesis kit commercially available from Applied Biosystems. Reverse transcription PCR was done by adding 10 *μ*L of RNA to the PCR master mix.

### 5.6. Gene Expression Analysis of CDK Genes Using Real-Time PCR

q-RT-PCR amplification was performed in the presence of the double-stranded DNA binding dye SYBR Green (Molecular Probes) and monitored in real time with the Opticon continuous fluorescence detection system. In quantification of the relative level of transcripts of CDKs genes, all reactions were taken in triplicate and results are shown as average of all the three reactions generated as graph in automated ABI 7500 SDS software of RT-PCR. Tubulin gene was taken as endogenous control. Normal root apical zone, elongation zone, and mature zone were considered as calibrator. Comparative analysis of CDKs gene expression level in apical, elongation, and mature zone of normal, endoreduplicated, and depolyploidized roots was generated using automated ABI 7500 real-time PCR. Accuracy of results was specified by the amplification plot and dissociation curve generated by automated ABI 7500 SDS software of RT-PCR.

### 5.7. Primers Used in Gene Expression Analysis

Primers used in quantification of the relative level of transcripts of CDKs genes were selected from the reprint of Miao et al. [30] [CDKA;1 and CDKA;2] and Andersen et al. [60] [tubulin *β*-2, CDKB2;1, CDKB2;2, CDKD1;1, and CDKD1;3] as listed in Table 1.

## Supplementary Material


**Supplementary data Figure 10:** shows changes in morphology of *Allium cepa* root growth after colchicine, NAA and BAP treatment. *Allium cepa* root grown in distilled water for 120h showed normal thin root. When young root (24h old) of *Allium cepa* was exogenously treated with NAA for 120h, C-tumor formation and inhibition in growth of root was observed. When young root was exogenously treated with BAP it showed faster and normal growth of root. When young root was treated with colchicine for 120h, c-tumor formation was observed. This colchicine treated root when treated with NAA for 120h showed no change in growth of root. But colchicine treated root when treated with BAP for 120h showed increase in growth of root.
**Supplementary data Figure: 11** Shows changes in length of *Allium cepa* roots after exogenous colchicine and NAA treatment.
**Supplementary data Figure: 12** Shows changes in length of *Allium cepa* roots after exogenous colchicine and BAP treatment.

## Figures and Tables

**Table 1:** Primers used in gene expression analysis.

Target gene	Organism	Orientation	Primer sequence
*TUBULIN*β*-2 *	*Arabidopsis thaliana *	Sense	GAGCCTTACAACGCTACTCTGTCTGTC
*TUBULIN*β*-2 *	*Arabidopsis thaliana *	Antisense	ACACCAGACATAGTAGCAGAAATCAAG
*CDKB2;1 *	*Arabidopsis thaliana *	Sense	TTTAGAGAGCGATGGACGAGG
*CDKB2;1 *	*Arabidopsis thaliana *	Antisense	AGCATTCGCAAAATGGAGAT
*CDKB2;2 *	*Arabidopsis thaliana *	Sense	AGAGATTGATAGAGATGGACAACAAT
*CDKB2;2 *	*Arabidopsis thaliana *	Antisense	AGGATCACGAGCGAGCATAC
Glyma*;CDKA;1 *	*Glycine max *	Sense	CTGAGGTGTCTTATTAGT
Glyma*;CDKA;1 *	*Glycine max *	Antisense	CGGGTAAATAGGTAACAA
Glyma*;CDKA;2 *	*Glycine max *	Sense	GATGGCAAACGTGTTTAT
Glyma*;CDKA;2 *	*Glycine max *	Antisense	ATGAAAAAGTAATATGCA
*Arath;CDKD1;1 *	*Arabidopsis thaliana *	Sense	CTGGGAATGGCGAAATCAAGGCATC
*Arath;CDKD1;1 *	*Arabidopsis thaliana *	Antisense	GTTGCTGATAGGTATCTAAAGCGAGAGGT
*Arath;CDKD1;3 *	*Arabidopsis thaliana *	Sense	GGATTTTCGCCTGCTGGGGCAAACCAGCGT
*Arath;CDKD1;3 *	*Arabidopsis thaliana *	Antisense	CAGCCAAAGAAAGTTGCTGATAGGTATCTC
