# Supplementary material for: Systemic Control of Cell Division and Endoreduplication by NAA and BAP by Modulating CDKs in Root Tip Cells of Allium cepa
Source: Biomed Res Int. 2014 May 18;2014:453707. doi: 10.1155/2014/453707 (PMC4052472; doi:10.1155/2014/453707)
Supplement: Supplementary file 1 — Supplementary data Figure 10: shows changes in morphology of Allium cepa root growth after colchicine, NAA and BAP treatment. Allium cepa root grown in distilled water for 120h showed normal thin root. When young root (24h old) of Allium cepa was exogenously treated with NAA for 120h, C-tumor formation and inhibition in growth of root was observed. When young root was exogenously treated with BAP it showed faster and normal growth of root. When young root was treated with colchicine for 120h, c-tumor formation was observed. This colchicine treated root when treated with NAA for 120h showed no change in growth of root. But colchicine treated root when treated with BAP for 120h showed increase in growth of root. Supplementary data Figure: 11 Shows changes in length of Allium cepa roots after exogenous colchicine and NAA treatment. Supplementary data Figure: 12 Shows changes in length of Allium cepa roots after exogenous colchicine and BAP treatment. [file 453707.f1.pdf]

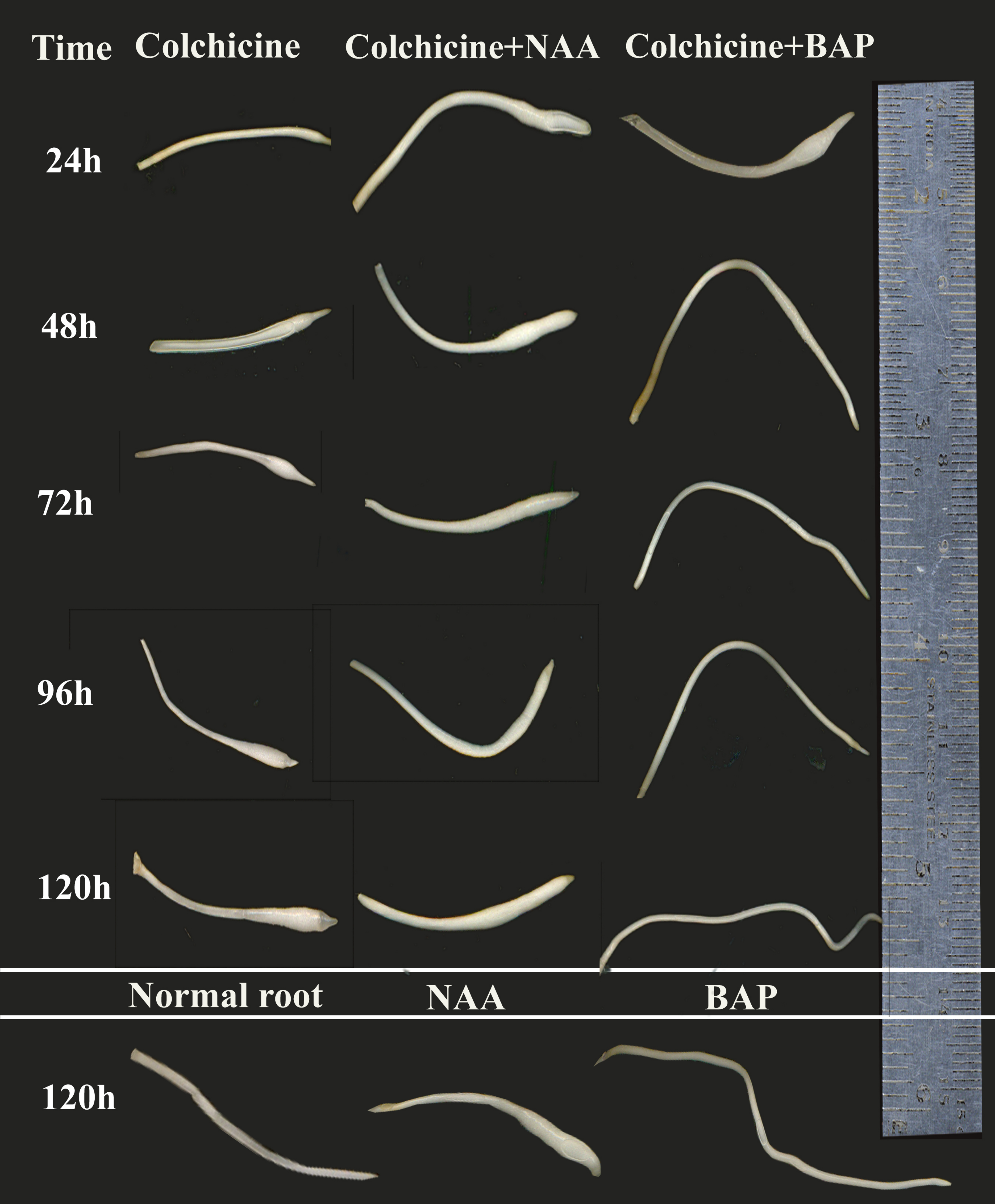

Supplementary data figure: 10 shows changes in morphology of roots after colchicine, NAA and BAP treatment

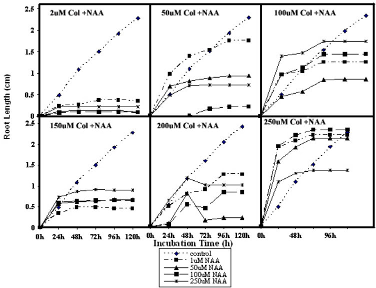

Supplementary data figure: 11 shows changes in length of endoreduplicated roots after NAA treatment

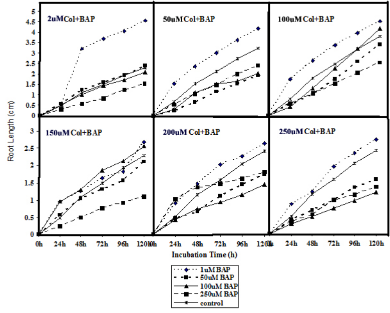

Supplementary data figure: 12 shows changes in length of endoreduplicated roots after BAP treatment
